# Variant Creutzfeldt-Jakob Disease Death, United States

**DOI:** 10.3201/eid1109.050371

**Published:** 2005-09

**Authors:** Ermias D. Belay, James J. Sejvar, Wun-Ju Shieh, Steven T. Wiersma, Wen-Quan Zou, Pierluigi Gambetti, Stephen Hunter, Ryan A. Maddox, Landis Crockett, Sherif R. Zaki, Lawrence B. Schonberger

**Affiliations:** * Centers for Disease Control and Prevention, Atlanta, Georgia, USA;; † Florida Department of Health, Tallahassee, Florida, USA;; ‡ Case Western Reserve University, Cleveland, Ohio, USA

**Keywords:** Creutzfeldt-Jakob disease, variant Creutzfeldt-Jakob disease, prion disease, transmissible spongiform encephalopathy, epidemiology, surveillance, public health, research

## Abstract

The only variant Creutzfeldt-Jakob disease (vCJD) patient identified in the United States died in 2004, and the diagnosis was confirmed by analysis of autopsy tissue. The patient likely acquired the disease while growing up in Great Britain before immigrating to the United States in 1992. Additional vCJD patients continue to be identified outside the United Kingdom, including 2 more patients in Ireland, and 1 patient each in Japan, Portugal, Saudi Arabia, Spain, and the Netherlands. The reports of bloodborne transmission of vCJD in 2 patients, 1 of whom was heterozygous for methionine and valine at polymorphic codon 129, add to the uncertainty about the future of the vCJD outbreak.

## Abstract 2

Reports of secondary bloodborne transmission of vCJD add to the uncertainty about the future of the vCJD outbreak.

Variant Creutzfeldt-Jakob disease (vCJD) was first reported in 1996 in the United Kingdom and has been causally linked to eating cattle products contaminated with the bovine spongiform encephalopathy (BSE) agent (*1**–**3*). Both vCJD and BSE are rapidly progressive neurodegenerative disorders classified as transmissible spongiform encephalopathies (TSEs) or prion diseases. TSEs are characterized by 1) incubation periods measured in years, 2) presence in the brain of an unconventional agent called a prion, 3) absence of inflammatory reaction, and 4) a neuropathologic feature consisting typically of spongiform lesions, astrogliosis, and neuronal loss. vCJD is distinguished from the more common TSE in humans, sporadic CJD, by the younger median age (28 years and 68 years, respectively) of the patients and by its clinical and neuropathologic manifestations.

In 2002, the likely occurrence of vCJD was reported in a 22-year-old woman living in Florida and represented the first detection of the disease in North America (*4*). In this report, we describe this patient's illness and provide an update on the epidemiologic features of the ongoing vCJD outbreak worldwide, including recent reports of bloodborne transmission of vCJD.

## Case Report

In early November 2001, the patient in Florida was evaluated for depression and memory loss that adversely affected her work performance and may have contributed to a traffic ticket she received for failure to yield the right of way at a traffic sign. In December 2001, the patient developed involuntary movements, gait disturbances, difficulty dressing, and incontinence. The following month, she was taken to a local emergency department; a computed tomographic scan of her brain showed no abnormalities, a diagnosis of panic attack was made, and antianxiety medications were prescribed.

In late January 2002, the patient was transported to the United Kingdom, where her mother resided. During the ensuing 3 months, the patient's motor and cognitive deficits worsened, which caused falls resulting in minor injuries; she had difficulty taking care of herself, remembering her home telephone number, and making accurate mathematical calculations. She also experienced confusion, hallucination, dysarthria, bradykinesia, and spasticity. An electroencephalogram evaluation showed no abnormalities, but a brain magnetic resonance imaging (MRI) study showed the characteristic signal abnormalities in the pulvinar and metathalamic regions suggestive of vCJD. Western blot and immunohistochemical analyses of tonsillar biopsy tissue demonstrated the presence of protease-resistant prion protein, which supported the diagnosis of vCJD. By September 2002, the patient was bedridden. An experimental treatment with quinacrine was given to the patient for 3 months, but she showed little improvement. She remained in a state of akinetic mutism and died in June 2004, ≈32 months after illness onset.

**Figure 1:**
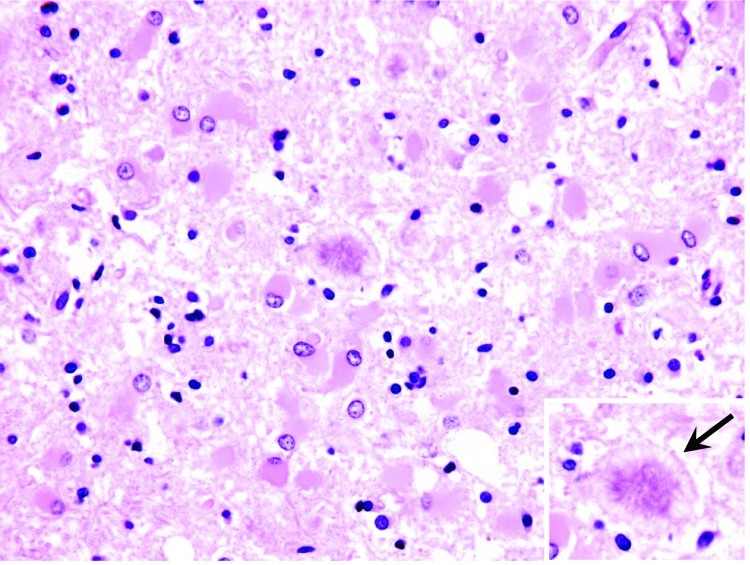
Histopathologic changes in frontal cerebral cortex of the patient who died of variant Creutzfeldt-Jakob disease in the United States. Marked astroglial reaction is shown, occasionally with relatively large florid plaques surrounded by vacuoles (arrow in inset) (hematoxylin and eosin stain, original magnification ×40).

**Figure 2:**
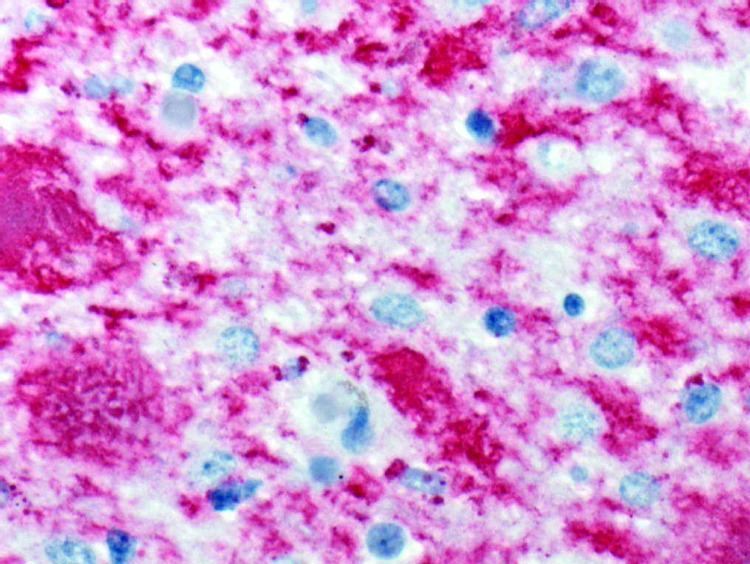
Immunohistochemical staining of cerebellar tissue of the patient who died of variant Creutzfeldt-Jakob disease in the United States. Stained amyloid plaques are shown with surrounding deposits of abnormal prion protein (immunoalkaline phosphatase stain, naphthol fast red substrate with light hematoxylin counterstain; original magnification ×158).

The patient was born in Great Britain in 1979 and immigrated to the United States with her family in 1992. She had no history of surgery or receipt of blood or blood products, and she was never a blood donor. Consistent with findings for vCJD patients in the United Kingdom associated with potential foodborne exposure, this patient was homozygous for methionine at polymorphic codon 129 of the prion protein gene. A full autopsy was performed, and neuropathologic examination of brain tissue showed the presence of florid plaques and severe cortical atrophy (Figure 1). Immunohistochemical staining for the prion protein showed numerous plaquelike formations along with a synaptic staining pattern similar to that previously described in other vCJD patients (Figure 2).

This patient is the only US resident with a confirmed diagnosis of vCJD. She was likely exposed to BSE while growing up in the United Kingdom from 1980 to 1992, which suggests an incubation period of 9–21 years (Table). The illness duration in this patient (≈32 months) was much longer than the median illness duration for patients in the United Kingdom with vCJD (14 months, range 6–40 months).

**Table:** Estimated range of incubation periods for variant Creutzfeldt-Jakob disease cases with presumed route and year of exposure*

Presumed route of exposure and country of residence†	Period of potential BSE/vCJD exposure	Estimated range of incubation period (y)
Foodborne		
Canada	1987–1990	11–14
Ireland	1989–1995	5–10
Japan‡	1989	12
United Kingdom (Leicestershire, England)	1980–1985	10–16
United States	1980–1992	9–21
Bloodborne		
United Kingdom	1996	6.5
United Kingdom§	1999	>5

*BSE/vCJD, bovine spongiform encephalopathy/variant Creutzfeldt-Jakob disease. †Estimated range of incubation periods are based on a single vCJD case except for Leicestershire, England, where the Leicestershire Health Authority reported the estimated incubation period for a cluster of 5 vCJD cases. ‡Patient was reported to have stayed in the United Kingdom ≈24 days. §Patient with presumed bloodborne transmission died of a nonneurologic disease. The presence of the vCJD agent was detected in the spleen and cervical lymph node; the patient was heterozygous for methionine and valine at the polymorphic codon 129 of the prion protein gene.

## Updates on vCJD

**Figure 3:**
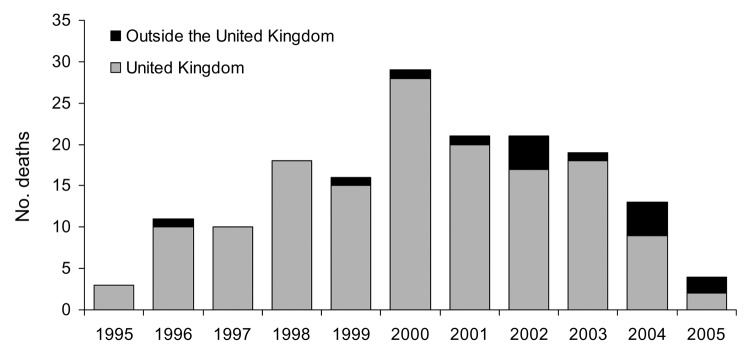
Number of deceased variant Creutzfeldt-Jakob disease patients worldwide (150 from the United Kingdom and 15 outside the United Kingdom) by year of death, June 2005.

As of early August 2005, 157 vCJD patients were reported from the United Kingdom: 13 have been reported from France, 3 from Ireland, and 1 each from Canada, Italy, Japan, Portugal, Spain, the Netherlands, and the United States (Figure 3). Similar to the vCJD patient from the United States, 1 patient from Ireland and the patients from Canada and Japan were likely exposed to the BSE agent during their residence in the United Kingdom. Preliminary information indicates that the Japanese patient spent only ≈24 days in the United Kingdom. In addition, the US National Prion Disease Pathology Surveillance Center confirmed a vCJD diagnosis by analyzing a brain biopsy sample from a 33-year-old Saudi man admitted to a hospital in Saudi Arabia. Although detailed information on this patient was not available, he may have visited the United Kingdom, if at all, only for several days. Thus, the patient likely contracted the disease in Saudi Arabia after eating BSE-contaminated cattle products imported from the United Kingdom.

Certain characteristics distinguishing vCJD from classic CJD raised early concerns about possible secondary bloodborne spread of vCJD, especially in light of the lack of experience with this newly emerged disease. Of specific concern was the detection of the agent in lymphoid tissues and the possibility of prionemia as the agent spreads from the gut to the brain. In 1997, to monitor for the possible bloodborne transmission of vCJD, researchers in the United Kingdom began investigating recipients of blood components obtained from donors who subsequently died of vCJD (*5*). As of February 2004, 48 recipients were identified, including 18 who had survived for >4 years after transfusion. Two of these 18 recipients had evidence of bloodborne transmission of vCJD. One of the 2 recipients was 62 years of age and had received 5 units of erythrocytes in 1996 (*5*). One of these units was traced to a 24-year-old donor in whom vCJD developed >3 years after the blood was donated. In 2002 (6.5 years after the transfusion), vCJD developed in the recipient, who died 13 months after illness onset. An autopsy confirmed the diagnosis of vCJD.

The second patient potentially linked with bloodborne transmission of vCJD was an elderly person who received a unit of erythrocytes in 1999. vCJD developed in the donor of the unit 18 months after blood was donated (*6*). The recipient died of a ruptured aortic aneurysm 5 years after the transfusion. Tests of the patient's spleen and cervical lymph node detected protease-resistant prion protein with a Western blot mobility pattern and glycoform ratio similar to those seen in other vCJD patients. These results and the absence of neurologic symptoms and brain pathologic findings indicated that this patient had a subclinical vCJD infection. Prion protein gene sequencing showed heterozygosity for methionine and valine at codon 129, which indicated that persons not homozygous for methionine (>50% of the population) can be infected by the vCJD agent.

In the United States, the risk of bloodborne transmission of vCJD is low because of the absence of indigenous vCJD and the geographic-based donor deferral policy instituted by the Food and Drug Administration in 1999. This policy excludes from donating blood in the United States persons who resided in or had extended visits to the United Kingdom or other European countries during periods of greatest concern for BSE exposure (*7*).

The exact incubation period for foodborne vCJD is unknown. However, a range of possible incubation periods was estimated for 4 vCJD patients who likely acquired the disease during their residence in the United Kingdom and for 5 vCJD patients reported as part of a cluster in Leicestershire, England (Table). The median of the estimated range of incubation periods for these 9 vCJD patients was 13 years. The incubation period for the vCJD patient linked to bloodborne transmission was shorter (6.5 years). This finding could be the result of direct transmission of the agent into the bloodstream and adaptation of the agent to the human population, thus reducing or eliminating the species barrier (Table).

## Conclusions

Patients with vCJD can be distinguished from patients with the more common sporadic CJD by their younger median age at death (28 years and 68 years, respectively), predominantly psychiatric manifestations at illness onset, delayed appearance of frank neurologic signs, absence of a diagnostic electroencephalographic pattern, presence of the pulvinar sign on MRI, and a longer median illness duration (<6 and 14 months, respectively) (*3**,**8*). Almost all vCJD patients have died before 55 years of age, compared with only ≈10% of sporadic CJD patients. The most striking early neurologic manifestation in some vCJD patients was painful sensory symptoms (dysesthesia or paresthesia). Other neurologic signs, such as chorea, dystonia, and myoclonus, commonly develop late in the course of vCJD. An MRI result with symmetrically increased signal intensity in the pulvinar region relative to the signal intensity in other deep and cortical gray mater areas has been reported in >75% of vCJD patients. The presence of this MRI feature, known as pulvinar sign, may suggest a vCJD diagnosis in the appropriate clinical context. A prominent, early involvement of lymphoid tissues has enabled a reasonably accurate premortem diagnosis of vCJD, using tonsillar biopsy. However, a more definitive diagnosis of vCJD may require testing autopsy brain tissues.

In June 2005, the US Department of Agriculture confirmed BSE in an ≈12-year-old cow born and raised in Texas. This is the first time an indigenous BSE case was detected in the United States. A previous BSE-positive cow identified in Washington State was imported from Canada (*3*) where, to date, 4 additional BSE cases have been identified. The identification of BSE in North America and the likelihood of bloodborne transmission of vCJD underscore the need to continue surveillance to monitor the occurrence of vCJD in the United States (*3*). The case-patient described in this report illustrates the need for physicians to remain vigilant for the possibility of vCJD in patients with the signs and symptoms described. Physicians should report suspected vCJD cases to local and state health departments. Because the clinical manifestation of vCJD can overlap that of sporadic CJD, brain autopsies should be sought in all suspected cases to establish the diagnosis and to help monitor the occurrence of vCJD and other potentially emerging forms of CJD. Free state-of-the art prion disease diagnostic testing is available from the National Prion Disease Pathology Surveillance Center (http://www.cjdsurveillance.com), which was established to facilitate autopsy performance and testing (*8*). Physicians are encouraged to use the services of the surveillance center to evaluate neuropathologic changes in their patients with suspected or clinically diagnosed prion disease.
